# Targeted Metabolomic Profiling of Emotional and Reflex Tears: A Paired Exploratory Study

**DOI:** 10.3390/metabo16090607

**Published:** 2026-08-25

**Authors:** Jiahua Liu, Xiqiao Gao, Hao Liang, Jiahao Ye

**Affiliations:** 1 Medical School, Hunan University of Chinese Medicine, Changsha 410208, China; 2 Academy of Chinese Medical Sciences, Hunan University of Chinese Medicine, Changsha 410208, China; 3 Institute of TCM Diagnosis, Hunan University of Chinese Medicine, Changsha 410208, China

**Keywords:** emotional tears, reflex tears, targeted metabolomics, paired study, machine learning

## Abstract

**Objectives:** Emotional tears are generated in a distinct neurophysiological context from reflex tears, but their metabolic composition remains poorly understood. **Methods:** This exploratory paired targeted-metabolomics study compared emotional and reflex tears collected from 22 healthy volunteers using a 600-multiple-reaction-monitoring platform. Among 412 detected metabolites, 344 were retained after data preprocessing. Paired statistical analysis prioritized 23 candidate metabolites based on the combined criteria of unadjusted *p*-value, fold change, and consistency of within-subject change. **Results:** Seven candidates were lower, and 16 were higher in emotional tears. Salicylic acid was retained in the descriptive candidate set but excluded from the primary machine-learning analysis because a contribution from the reflex-tear induction procedure could not be ruled out. Among the remaining 22 candidates, 4-hydroxy-3-methylbenzoic acid, vanillic acid, cytidine-5′-monophosphate, and hydroxyphenyllactic acid were consistently ranked among the leading features. A fixed four-metabolite combination achieved an area under the receiver operating characteristic curve of 0.864 (95% CI, 0.756–0.957) under ordinary leave-one-subject-out cross-validation. When candidate screening, feature selection, and model fitting were repeated within each training fold, the best nested pipeline achieved an area under the curve of 0.725 (95% CI, 0.603–0.843). Pathway mapping further linked the candidate metabolites to histidine, tyrosine, fatty-acid, ether-lipid, and ubiquinone-related metabolism. **Conclusions:** These findings demonstrate measurable within-subject metabolic differences between emotional and reflex tears and identify a focused set of candidate metabolites for future validation. Because no individual metabolite remained significant after false discovery rate correction and no independent validation cohort was available, the candidate signals and discrimination models should be confirmed in larger, independently collected cohorts.

## 1. Introduction

Emotional tearing is a distinctive form of lacrimal secretion, yet its molecular composition has received far less attention than that of other body fluids. Tears are easy to collect and do not require invasive procedures, which makes them attractive for biomarker research [1]. They contain proteins, lipids, and a broad range of small molecules. Their composition is shaped by ocular-surface conditions and may also carry information about wider physiological or pathological processes [2]. This has encouraged growing interest in tear fluid as a source of noninvasive biomarkers. Emotional tears, however, remain comparatively poorly characterized, and their biological meaning is still uncertain [3,4].

Crying in response to emotion is not simply an increase in tear volume. Sadness and other affective states engage central neural circuits, autonomic pathways, endocrine responses, and the lacrimal glands. The resulting tear secretion may therefore reflect part of the physiological response to emotion, including changes related to stress, neural activity, and metabolism [5]. Studying small molecules in emotional tears may help clarify how this type of secretion is generated and whether any of its molecular features can serve as objective indicators of emotional state.

Tear fluid is also heterogeneous in physiological origin. Basal tears maintain lubrication and ocular-surface homeostasis. Reflex tears are produced mainly as a protective response to external irritation. Emotional tears arise in a different setting, namely affective processing, and are likely to involve a more complex combination of neural and endocrine regulation. Thus, although both emotional crying and external stimulation increase tear secretion, the biological context is not the same. Emotional tearing is linked more closely to affective and neuroendocrine activity, whereas reflex tearing mainly serves a protective role at the ocular surface [6]. Comparing the two within the same individuals offers a practical way to look for molecular features associated more specifically with emotional tearing.

Metabolomics is well suited to this comparison because it captures small-molecule variation across biological samples and can reveal signatures linked to particular physiological states [7]. Targeted approaches based on multiple reaction monitoring (MRM) offer advantages in quantitative consistency, sensitivity, and reproducibility. These features are particularly useful when sample volume is limited, as is often the case for tear fluid. Dammeier et al., for example, used targeted metabolomics in tears and reported intra-individual coefficients of variation below 20% for most analytes, with 60 metabolites detected consistently across all samples [8]. Such findings support the use of targeted metabolomics for a more focused assessment of metabolic differences among tear types. However, important gaps remain. Previous molecular studies of emotional tears have focused mainly on proteomic differences or comparisons between tears produced under different emotional conditions. Direct paired metabolomic comparisons of emotional and reflex tears collected from the same individuals remain scarce. Moreover, few studies have examined whether exploratory metabolite patterns retain discriminatory value when multiple-testing uncertainty and the optimistic bias introduced by feature screening in a small dataset are explicitly considered. A within-subject targeted-metabolomics design, combined with conservative statistical interpretation and nested validation, may therefore provide a more rigorous initial assessment of metabolic differences associated with emotional tearing.

We therefore conducted an exploratory paired study using a 600-MRM targeted metabolomics platform to compare emotional and reflex tears collected from the same healthy volunteers. The study had three aims: to characterize the metabolic profiles of the two tear types, to identify exploratory candidate metabolites with potential discriminatory value, and to evaluate small metabolite combinations using participant-level and strict nested leave-one-subject-out cross-validation. This design enabled an initial characterization of tear-type metabolic differences and the prioritization of candidate metabolites and multivariable patterns for subsequent validation.

## 2. Methods

### 2.1. Study Participants

The Ethics Committee of Jili Hospital approved this paired study (approval no. 2021-04; 27 May 2021). The study was also registered in the Chinese Clinical Trial Registry (ChiCTR2100047025). Every participant provided written informed consent before enrollment. Healthy young volunteers were recruited from graduate students at Hunan University of Chinese Medicine. No formal a priori sample-size calculation was performed because reliable effect-size estimates for this specific paired metabolomic comparison were unavailable. The sample size was therefore based on the exploratory nature of the study and the feasibility of paired tear collection.

Baseline data included age, sex, uncorrected or corrected visual acuity, refractive status, bilateral tear-film breakup time, Schirmer I test results, ocular medication history, and contact-lens use. Participants had not recently taken drugs known to alter tear secretion or emotional state. Artificial tears, anti-inflammatory eye drops, and other topical ophthalmic drugs were not permitted during the week before sampling.

Participants were eligible if they were 17–35 years old, had no obvious organic disease on a recent routine health examination, had generally normal ocular findings with an absolute spherical equivalent <6.00 D, and had no systemic disease or acute illness such as fever or upper respiratory infection at the time of sampling. They also had to be able to complete film viewing and tear collection and to provide written informed consent.

Exclusion criteria were an absolute spherical equivalent ≥6.00 D; upper respiratory infection, conjunctivitis, or other mucosal inflammation during the previous 7 days; ocular surgery within 6 months; fever within 7 days; a history of psychiatric disorders such as depression or schizophrenia; recent major psychological trauma; chronic insomnia lasting more than 1 month; smoking; or excessive alcohol consumption.

All volunteers underwent routine ophthalmic examination. Twenty-two met the study criteria, completed both tear collections, and were included in the final analysis.

### 2.2. Tear Collection

Sampling was carried out between 16:00 and 21:00. Participants were required to have slept for at least 6 h in the preceding 24 h and could not wear contact lenses during collection. Before sampling, they washed their faces and removed cosmetics from the eyelids, eyelashes, and nearby facial skin.

Schirmer strips were used for collection. Once the first tear appeared at the eyelid margin, the strip was bent 90° at the marked notch and placed in the conjunctival sac at the junction of the lateral and middle thirds of the lower lid. Wetting length was read directly from the graduated strip.

Three strips were collected for each tear type from every participant, and each strip had to reach a wetted length >25 mm. The three strips obtained from one participant under the same condition were pooled and treated as one analytical sample. Strips were transferred to sterile 2.0 mL cryovials immediately after collection and stored at −80 °C. The two tear-collection sessions were separated by at least 24 h. The order of emotional and reflex tear collection was randomized using a computer-generated sequence.

### 2.3. Definition and Induction of Tear Types

Reflex tears (F group) were induced with external stimulation. A topical medicated oil (Fengyoujing) was applied to the infraorbital skin without allowing direct contact with the ocular surface, and collection began after tearing started. The exact brand, batch, and full composition of the stimulant used in the original experiment could not later be verified. Therefore, the possibility that exogenous components of the stimulant influenced tear metabolite profiles cannot be completely excluded. Salicylic acid was considered the most likely directly related candidate because of its potential association with methyl salicylate-containing formulations and was therefore excluded from the primary machine-learning analysis. However, we acknowledge that other unidentified metabolite alterations related to the induction procedure may also have occurred.

Emotional tears (Q group) were induced by a standardized sad film and collected as soon as visible tearing occurred. Both tear types were obtained from the same participants to limit between-person variability.

### 2.4. Sample Preparation

Frozen strips were thawed on ice. Each sample was placed in a 2 mL microcentrifuge tube, and 1500 μL of extraction solvent pre-cooled to −40 °C was added. The solvent contained methanol, acetonitrile, and water at 2:2:1, together with internal standards.

Samples were vortexed, sonicated in an ice-water bath for 15 min, vortexed again, and kept at −40 °C for 2 h. After centrifugation at 12,000 rpm for 15 min at 4 °C, 1200 μL of supernatant was transferred to a clean tube and dried.

The residue was reconstituted in 120 μL acetonitrile/water (6:4, *v*/*v*), vortexed, sonicated in an ice-water bath for 30 s, and centrifuged again at 12,000 rpm for 15 min at 4 °C. A 70-μL aliquot of the final supernatant was transferred to an autosampler vial for LC–MS/MS analysis.

### 2.5. LC–MS/MS Analysis

Metabolites were measured on an Agilent 1290 UHPLC system (Agilent Technologies (Santa Clara, CA, USA)) coupled to a SCIEX Triple Quad 6500+ triple-quadrupole mass spectrometer (SCIEX (Framingham, MA, USA)). Separation was performed on a Waters Atlantis Premier BEH Z-HILIC column (Waters Corporation (Milford, MA, USA)) (1.7 μm, 2.1 mm × 150 mm). Mobile phase A was ultrapure water/acetonitrile (9:1, *v*/*v*) with 10 mmol/L ammonium acetate; mobile phase B was acetonitrile/ultrapure water (9:1, *v*/*v*) with the same concentration of ammonium acetate. The autosampler was kept at 4 °C, and the injection volume was 1 μL.

Data were acquired in multiple reaction monitoring (MRM) mode. Source settings were as follows: curtain gas, 35 psi; ion-spray voltage, +5500 V in positive mode and −4500 V in negative mode; source temperature, 400 °C; and ion source gases 1 and 2, 50 psi each.

### 2.6. Data Preprocessing and Statistical Analysis

The initial dataset contained 44 samples and 412 metabolites. Metabolites that were entirely missing in either tear condition or missing in more than half of all samples were excluded. Remaining missing values were imputed using one-half of the minimum observed value for the corresponding metabolite, leaving 344 metabolites for statistical analysis.

Because each participant contributed both emotional- and reflex-tear samples, all between-condition comparisons accounted for the paired study design. Two-sided paired *t*-tests were used to evaluate within-subject differences between emotional and reflex tears. Multiple testing across the 344 metabolites was controlled using the Benjamini–Hochberg false discovery rate procedure. Given the small sample size and exploratory nature of the study, the analyses were intended to estimate metabolic differences and prioritize candidate signals rather than establish definitive biomarker associations.

No metabolite remained statistically significant after FDR correction. Candidate selection was therefore treated as an exploratory, hypothesis-generating procedure. A metabolite was retained as an exploratory candidate only when it simultaneously met the following three criteria: an unadjusted paired-test *p*-value < 0.10, a geometric mean fold change for emotional versus reflex tears greater than 1.5 or less than 0.67, and a concordant direction of within-subject change in at least 16 of the 22 paired participants.

The unadjusted *p* < 0.10 threshold was used solely as a permissive candidate-retention criterion. In this small paired cohort, it was intended to reduce the likelihood of prematurely excluding potentially informative signals before their effect magnitude and within-subject consistency were considered. It was not interpreted as evidence of statistical significance, which was assessed using Benjamini–Hochberg-adjusted *p*-values.

The requirement for concordant change in at least 16 of 22 pairs corresponded to 72.7% of participants and represented an approximately three-quarter majority of the cohort. This pragmatic consistency criterion was used to prioritize metabolites showing broadly reproducible within-subject changes and to reduce the influence of differences driven by only a small number of participants or extreme observations. It was not intended as a formal significance threshold or a validated clinical cutoff.

Twenty-three metabolites met all three exploratory criteria. To evaluate the robustness of the candidate-level findings to deviations from normality, the distributions of the within-subject differences, defined as emotional tears minus reflex tears (Q−F), were assessed for the 23 exploratory candidate metabolites using Shapiro–Wilk tests and quantile–quantile plots. Two-sided paired t tests were retained as the primary analysis because the principal effect estimate was the mean paired difference and its 95% confidence interval. Given the small sample size and potential deviations from normality, two-sided Wilcoxon signed-rank tests were additionally performed as sensitivity analyses. These sensitivity analyses did not redefine the primary analysis or alter the exploratory candidate list. Salicylic acid remained in the exploratory candidate list; however, because the reflex-tear induction procedure could have contributed to this signal, it was excluded from the primary machine-learning analyses.

### 2.7. Exploratory Machine-Learning Prioritization of Candidate Metabolites

After salicylic acid was removed, 22 metabolites were evaluated by machine learning. We used LASSO logistic regression, linear support vector machine–recursive feature elimination (SVM-RFE), and random forest for feature selection and performance assessment.

Cross-validation was performed at the participant level: the emotional and reflex samples from the same person were always kept together. Ordinary leave-one-subject-out cross-validation (LOSO-CV) was used to examine discrimination within the exploratory feature set defined using the full dataset and for fixed metabolite combinations.

A limitation of this ordinary LOSO-CV was that the initial candidate screening had already used the full dataset, so some information from the held-out participant had entered the feature-definition step. To address this, we ran a strict nested LOSO-CV as a sensitivity analysis. In every outer fold, both samples from one participant were set aside, and candidate screening, salicylic acid exclusion, feature ranking, feature selection, model fitting, and threshold choice were repeated using only the remaining participants.

We used the nested LOSO-CV result as the more conservative estimate because it evaluated the full selection-and-modeling procedure rather than only a fixed feature set.

## 3. Results

### 3.1. Participant Characteristics

Twenty-two healthy volunteers completed the study. Because each person contributed one emotional and one reflex tear sample, the final dataset contained 44 samples, 22 in each condition. Mean age was 24.6 ± 4.1 years. Ten participants were male (45.5%), and 12 were female (54.5%). Fourteen participants (63.6%) were myopic, and the mean spherical equivalent was −2.75 ± 1.68 D.

Best-corrected visual acuity was normal bilaterally in every participant. Routine ophthalmic examination showed no clinically relevant abnormality, and there was no evidence of dry eye, conjunctival hyperemia, corneal epithelial injury, or active ocular inflammation. None had recent ocular surgery, contact lens-related complications, or pre-sampling use of medication known to affect tear secretion (Table 1).

### 3.2. Quality Control of the Metabolomic Data

**Figure 1:**
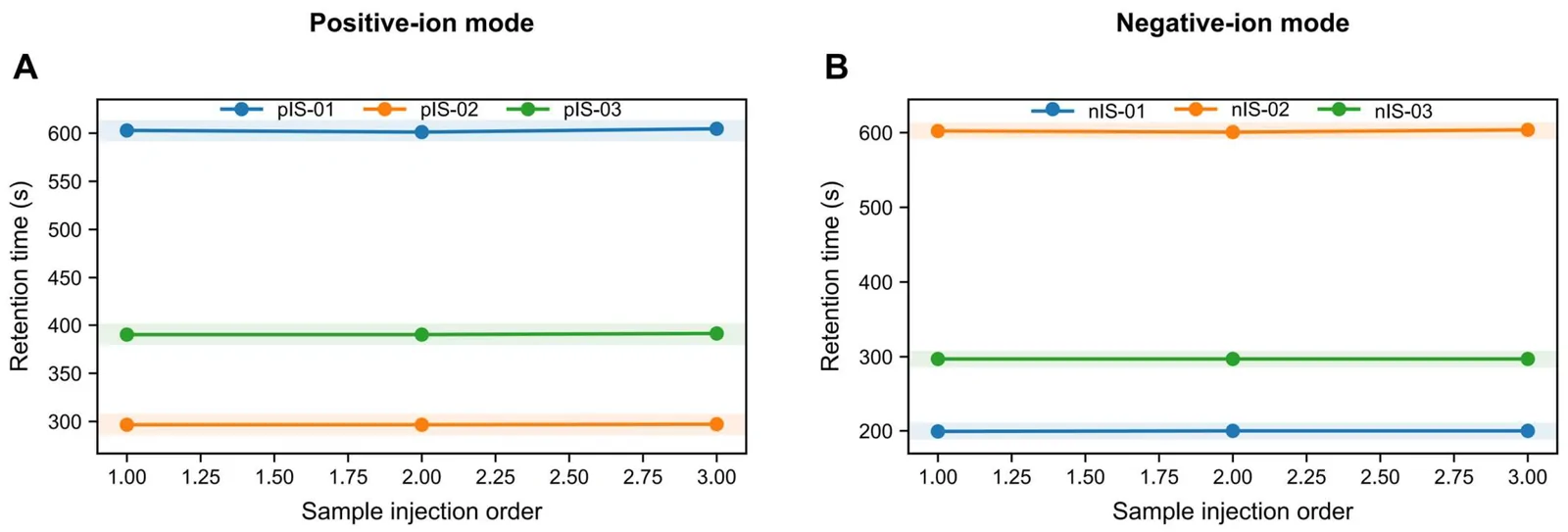
Quality control of internal-standard retention times in positive- and negative-ion modes. Legend: (**A**) Quality control of internal-standard retention times in positive-ion mode. (**B**) Quality control of internal-standard retention times in negative-ion mode. The x-axis indicates the sample injection order, and the y-axis indicates the retention time of the internal standards. The limited variation in retention times across samples indicates stable instrument performance and consistent chromatographic retention behavior.

**Figure 2:**
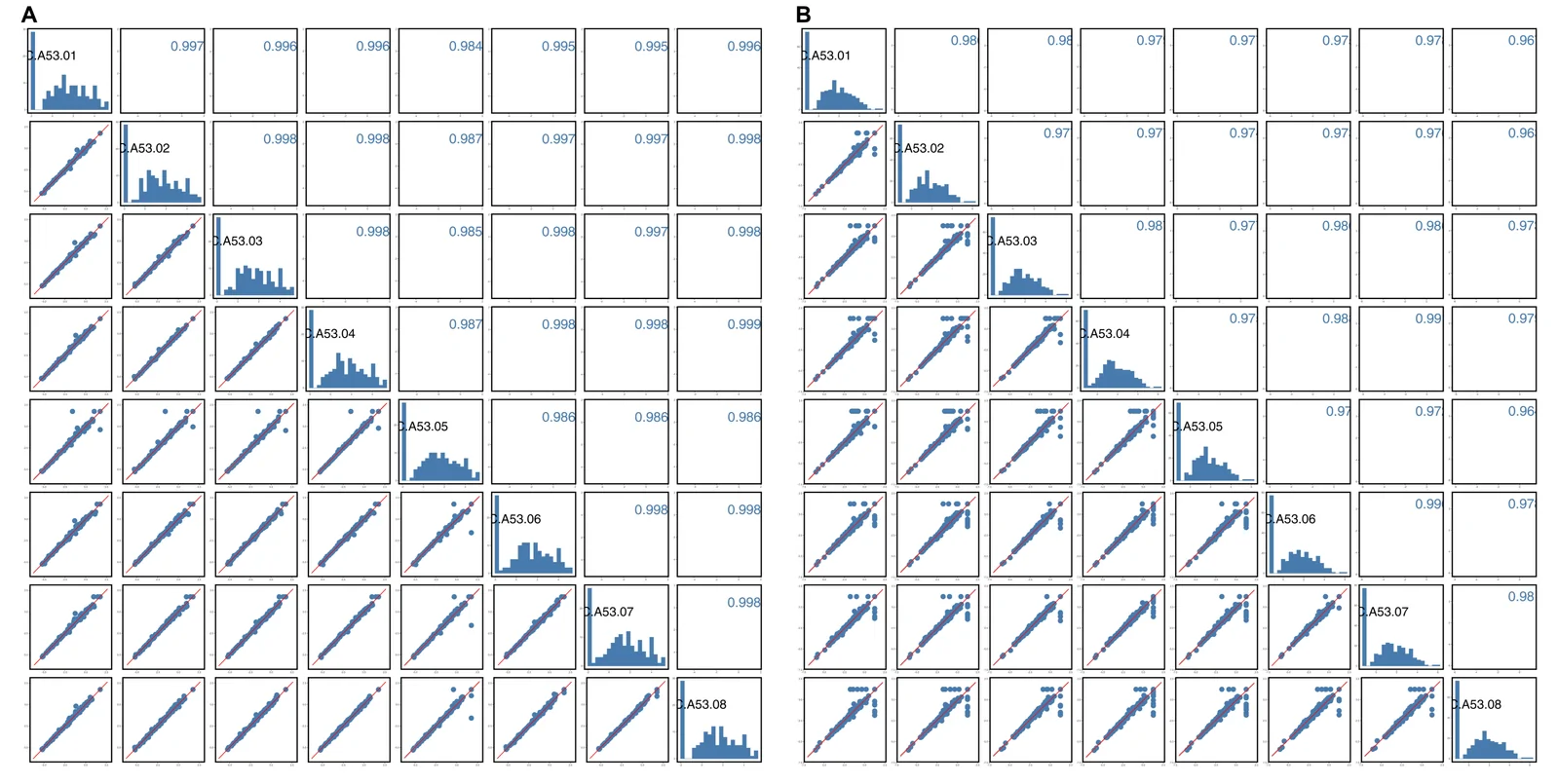
Correlation analysis of quality-control samples. Legend: (**A**) Correlation matrix of QC samples analyzed using reversed-phase chromatography (RP). (**B)** Correlation matrix of QC samples analyzed using hydrophilic interaction liquid chromatography (HILIC). QC samples were prepared by pooling equal aliquots of all study samples and were used to assess analytical stability and reproducibility throughout the acquisition sequence. Correlation coefficients approaching 1 indicate greater agreement among QC samples and higher analytical stability. The high correlations observed among QC samples indicate good overall data quality and reproducibility.

Analytical quality was checked from internal-standard retention times, correlations among quality-control (QC) samples, and calibration curves. Observed retention times stayed close to their expected values in both positive- and negative-ion modes, with only limited deviation across injections (Figure 1). QC samples were made by pooling equal aliquots of all study samples and were injected at intervals during the analytical sequence. Correlations among QC runs were high, indicating little signal drift over the acquisition period and good reproducibility of the platform (Figure 2). Internal-standard response stability in quality-control samples was further evaluated using the relative standard deviation (RSD, equivalent to the coefficient of variation). Across the RP and HILIC platforms, internal-standard RSD values ranged from 0.83% to 16.65%, indicating stable analytical performance during data acquisition (Table S1).

### 3.3. Metabolic Differences Between Emotional and Reflex Tears

The raw data consisted of 44 samples and 412 metabolites; 344 metabolites remained after missing-value filtering and imputation. Unsupervised PCA was first used to inspect overall variation. Emotional and reflex tear samples showed partial separation in principal-component space, but substantial overlap was still present (Figure S1).

Because the cohort was small and the design was paired, candidate screening relied mainly on paired tests, fold change, and consistency of change within participants. OPLS-DA was not used as the primary basis for feature selection and is reported only as a supplementary analysis (Figure S2).

Twenty-three metabolites satisfied all three exploratory criteria. Seven were lower in emotional tears: cytidine-5′-monophosphate, 4-hydroxy-3-methylbenzoic acid, glycerophosphocholine, salicylic acid, O-phosphoethanolamine, vanillic acid, and docosahexaenoic acid. The other 16 were higher in emotional tears: phenylethylamine, formiminoglutamic acid, kynurenic acid, citramalic acid, myristic acid, N6-acetyl-lysine, hydroxyphenyllactic acid, palmitoleic acid, methionine sulfoxide, p-cresol sulfate, quinic acid, 3,4-dihydroxyphenylacetic acid, methylimidazoleacetic acid, imidazolepropionic acid, myristoleic acid, and N-acetylglutamine.

Salicylic acid met the exploratory screening criteria, but the way reflex tears were induced left open the possibility of an external contribution to this signal. We therefore kept it in the full candidate list but did not regard it as an endogenous marker of emotional tears and excluded it from the primary machine-learning analysis.

**Figure 3:**
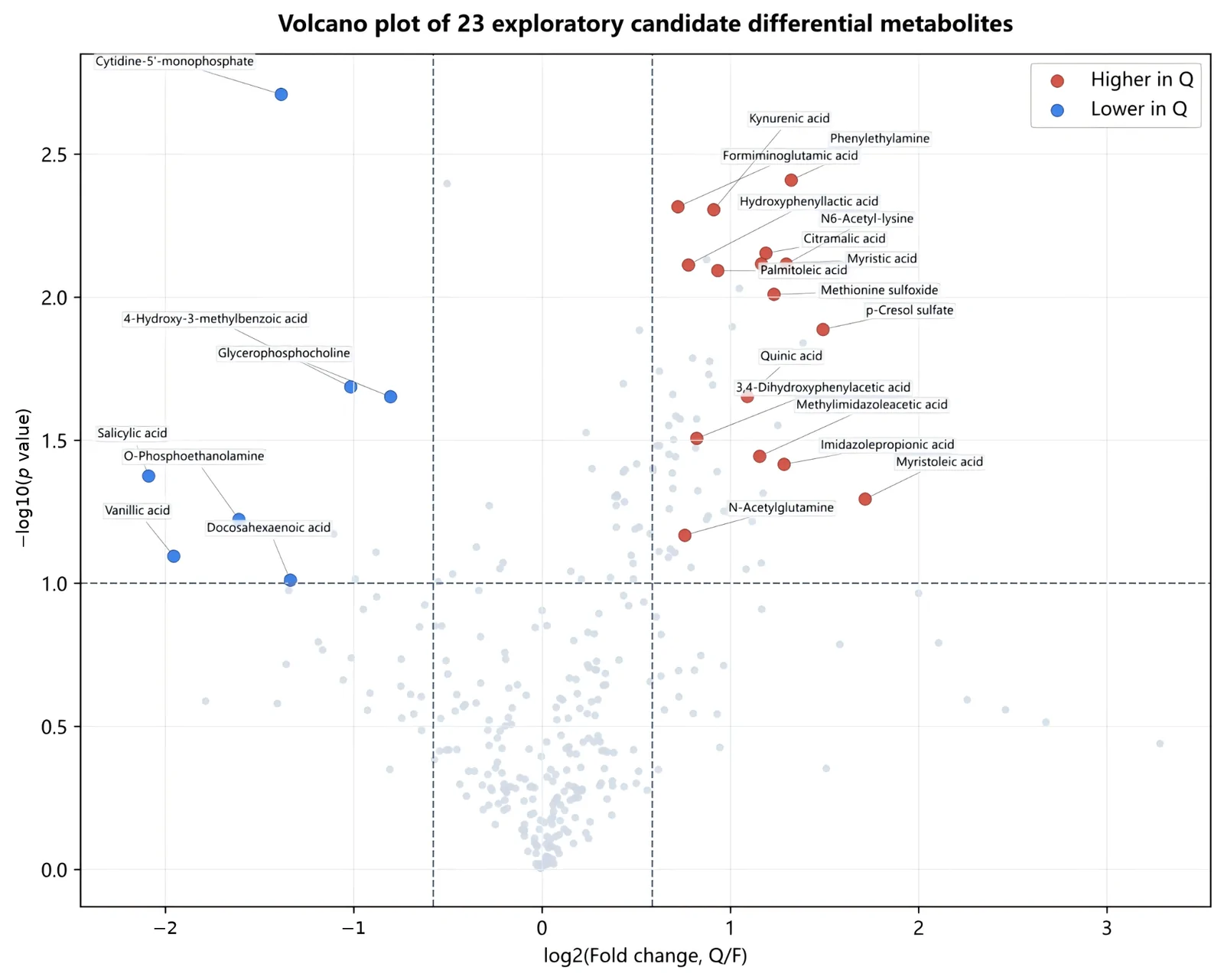
Volcano plot of 23 exploratory candidate metabolites in emotional and reflex tears. Legend: The x-axis represents the log2 fold change in emotional tears relative to reflex tears [log2(fold change, Q/F)], and the y-axis represents the −log10-transformed *p*-value from the paired statistical test. The 23 exploratory candidate metabolites are highlighted. Metabolites on the right were relatively higher in emotional tears, whereas those on the left were relatively lower. Candidate selection was based on paired *p*-values, fold change, and consistency of within-subject direction of change.The vertical dashed lines indicate the fold-change thresholds of 0.67 and 1.5 [log2(0.67) and log2(1.5), respectively], and the horizontal dashed line indicates the unadjusted *p*-value threshold of 0.10 [−log10(0.10) = 1].

**Figure 4:**
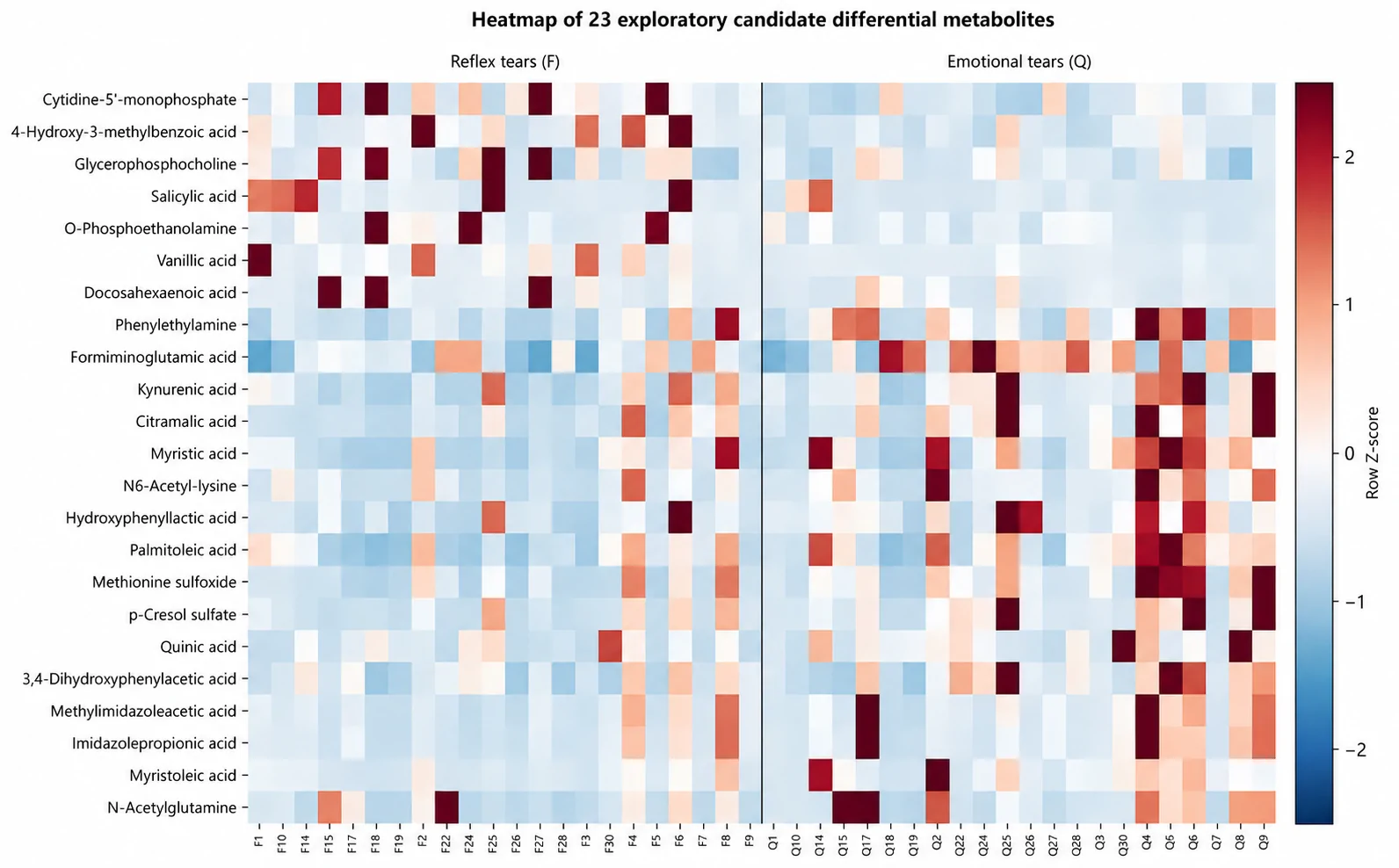
Hierarchical clustering heatmap of 23 exploratory candidate metabolites in emotional and reflex tears. Rows represent the 23 exploratory candidate metabolites, and columns represent tear samples. Colors indicate relative metabolite abundance after Z-score normalization, with warmer colors indicating higher relative abundance and cooler colors indicating lower relative abundance. Hierarchical clustering was used to visualize overall patterns and clustering tendencies of candidate metabolites across samples. The vertical black line separates reflex tear samples (F, **left**) from emotional tear samples (Q, **right**).

**Figure 5:**
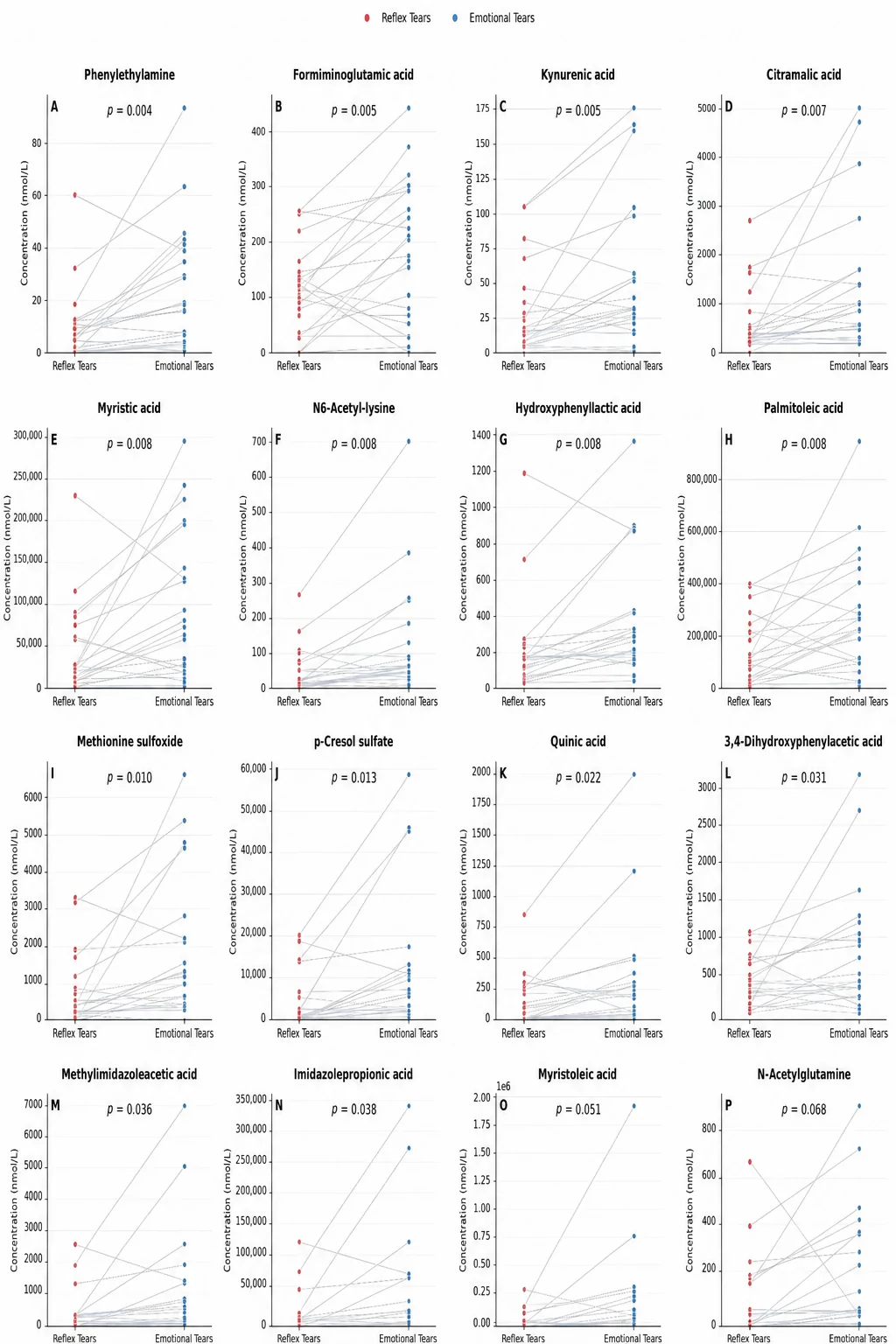
Paired distributions of exploratory candidate metabolites with higher abundance in emotional tears.Legend: Emotional- and reflex-tear measurements obtained from the same participant are connected by a line (n = 22 paired participants). Individual points represent single tear samples. Displayed *p*-values are unadjusted two-sided paired *t*-test *p*-values. None of the displayed metabolites remained statistically significant after Benjamini–Hochberg false discovery rate correction.

**Figure 6:**
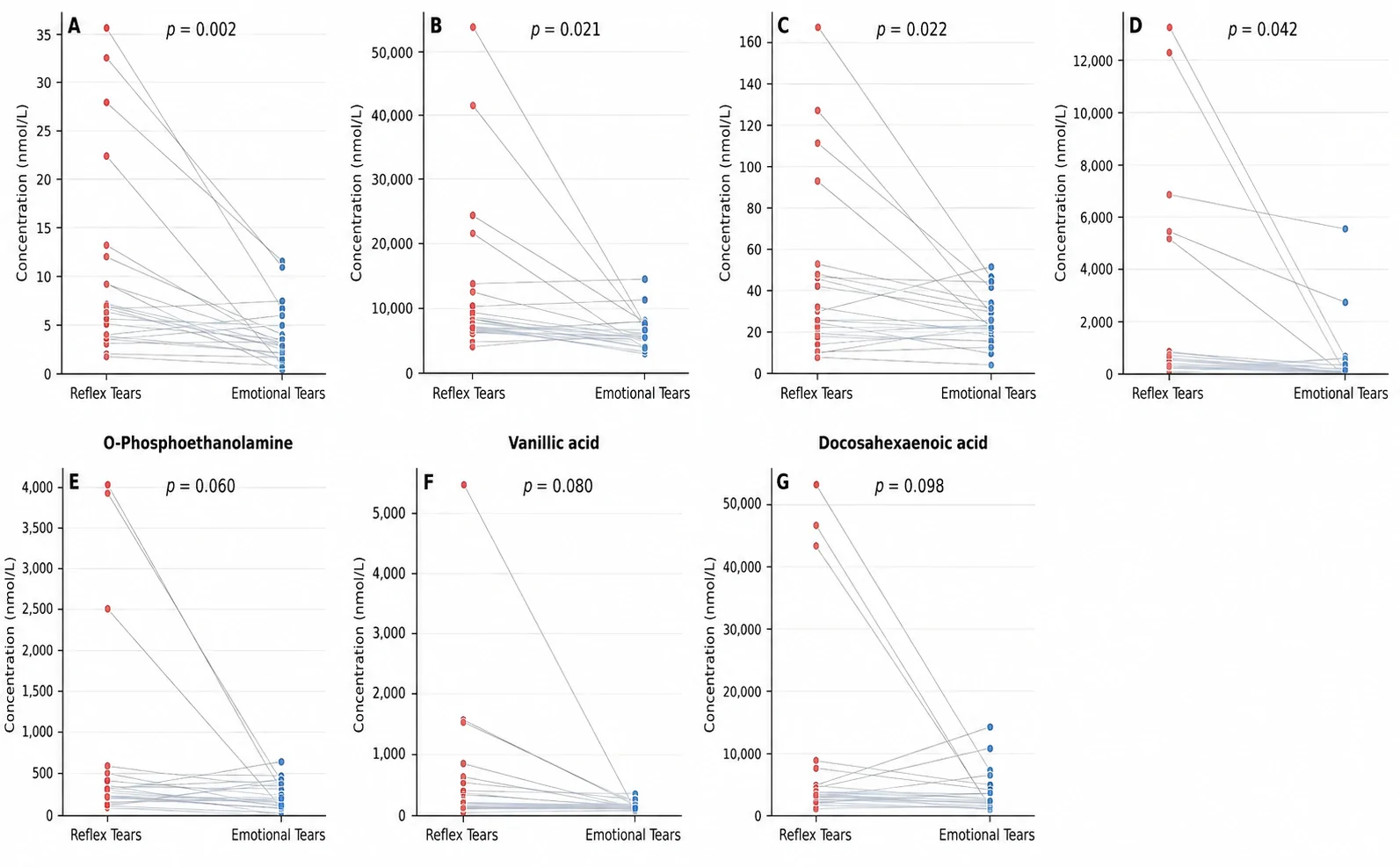
Paired distributions of exploratory candidate metabolites with lower abundance in emotional tears. Legend: Emotional- and reflex-tear measurements obtained from the same participant are connected by a line (n = 22 paired participants). Individual points represent single tear samples. Displayed *p*-values are unadjusted two-sided paired *t*-test *p*-values. None of the displayed metabolites remained statistically significant after Benjamini–Hochberg false discovery rate correction.

Heatmaps and paired point-and-line plots were used to visualize the distributions and within-subject changes in the 23 exploratory candidate metabolites across the two tear conditions. Detailed statistical information, including fold changes, paired mean differences with 95% confidence intervals, unadjusted paired *t*-test *p*-values, and Benjamini–Hochberg-adjusted *p*-values for all 23 exploratory candidate metabolites, is provided in Table S2. Wilcoxon signed-rank sensitivity analyses preserved the direction of change for all 23 exploratory candidates. Unadjusted Wilcoxon *p*-values were <0.05 for 22 of the 23 candidates, whereas docosahexaenoic acid showed weaker nominal evidence under the nonparametric analysis (*p* = 0.137). Several metabolites changed in a fairly consistent direction within participants, although marked person-to-person variation was also evident (Figure 3, Figure 4, Figure 5 and Figure 6).

### 3.4. Pathway Analysis of Exploratory Candidate Metabolites

Of the 23 exploratory candidate metabolites, 22 had valid KEGG annotations and were included in the pathway analysis. N-Acetylglutamine was excluded because no valid KEGG annotation was available, whereas docosahexaenoic acid was retained using KEGG identifier C06429. The pathway analysis was performed solely to generate biological hypotheses. Because the input metabolites were selected using unadjusted exploratory criteria and none remained statistically significant after metabolite-level FDR correction, the pathway-level findings were not considered confirmatory evidence of pathway activation or mechanistic involvement.

**Figure 7:**
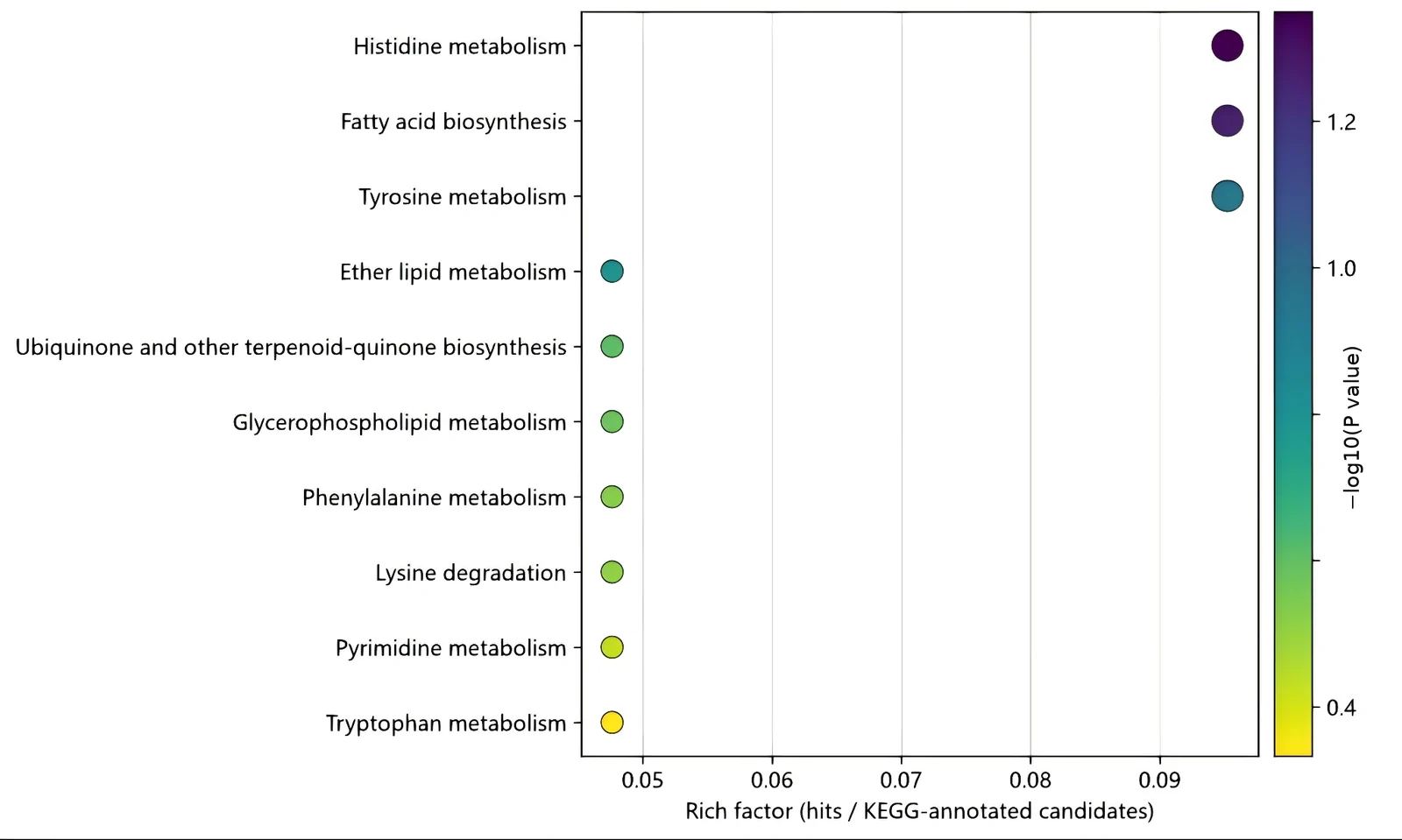
Exploratory KEGG pathway mapping of 22 annotated candidate metabolites in emotional and reflex tears. Legend: Of the 23 exploratory candidate metabolites, 22 with valid KEGG annotations were included in the pathway analysis; N-acetylglutamine was excluded because no valid KEGG annotation was available. Each bubble represents a KEGG pathway. The y-axis shows the pathway name, and the x-axis shows the enrichment factor. Bubble color represents the −log10-transformed unadjusted pathway-enrichment *p*-value, and bubble size represents the number of candidate metabolites mapped to each pathway. Because the input metabolites were selected using exploratory criteria and none remained significant after metabolite-level FDR correction, these pathway results should be interpreted as descriptive and hypothesis-generating rather than as evidence of pathway activation or mechanistic involvement.

The annotated candidates mapped to pathways including histidine metabolism, fatty acid biosynthesis, tyrosine metabolism, ether lipid metabolism, and ubiquinone and other terpenoid-quinone biosynthesis. Several pathways were represented by only a small number of candidate metabolites, further limiting the robustness of the enrichment results (Figure 7).

**Figure 8:**
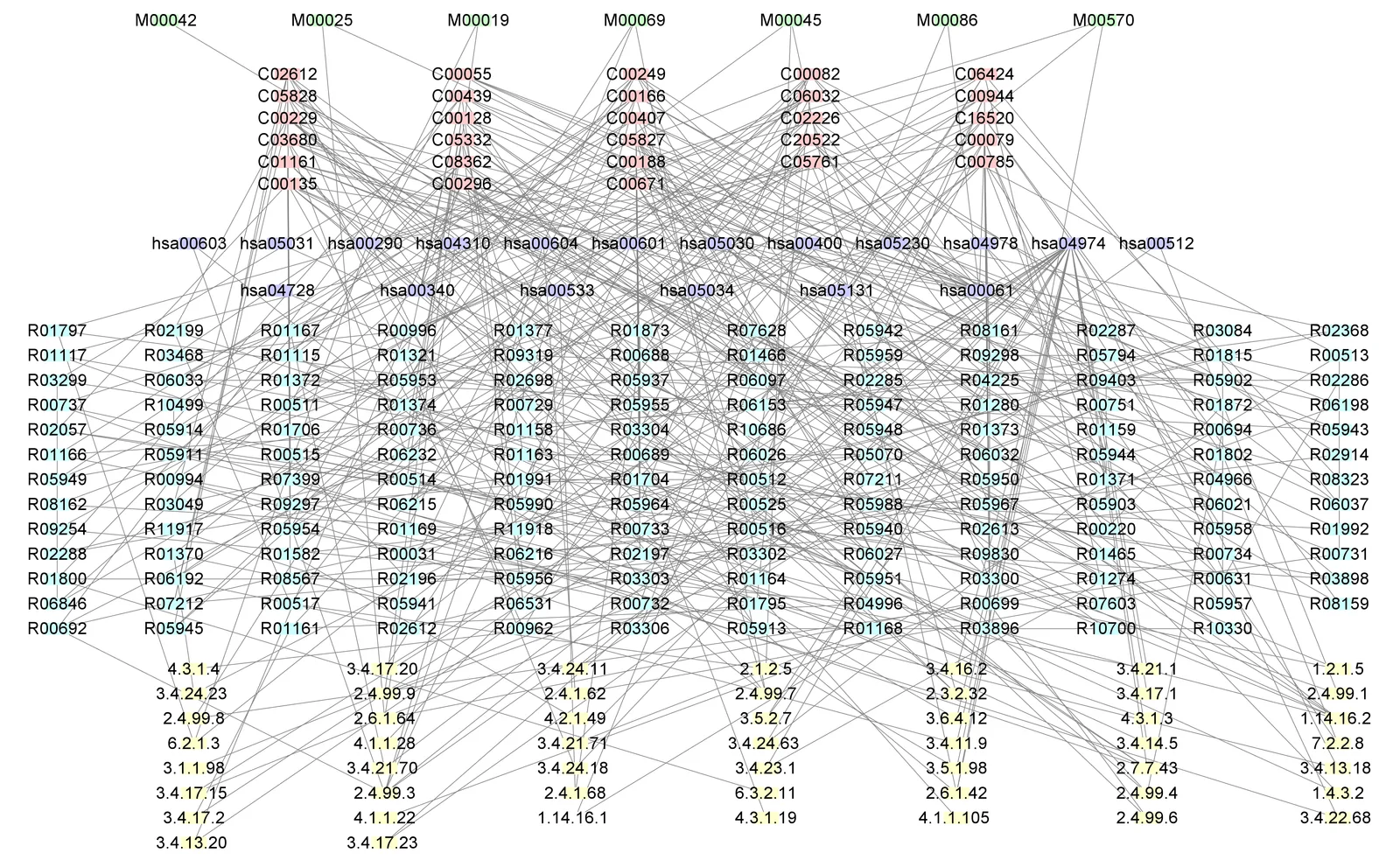
Exploratory metabolic network mapping of candidate metabolites in emotional and reflex tears. Legend: Cyan nodes represent reactions, yellow nodes represent enzymes, green nodes represent modules, purple nodes represent pathways, and red nodes represent compounds. The network is presented solely as a descriptive database-based visualization of relationships among the exploratory candidate metabolites and should not be interpreted as evidence of pathway activation, causal relationships, or mechanistic involvement.

Network mapping placed the candidates across histidine-related, fatty-acid, tyrosine-related, and membrane-lipid metabolic networks, with overlap among several pathways. These patterns provide descriptive biological context for the observed metabolic differences but do not establish that the mapped pathways were activated or directly involved in emotional tear formation (Figure 8).

### 3.5. Descriptive Single-Metabolite ROC Analyses

**Figure 9:**
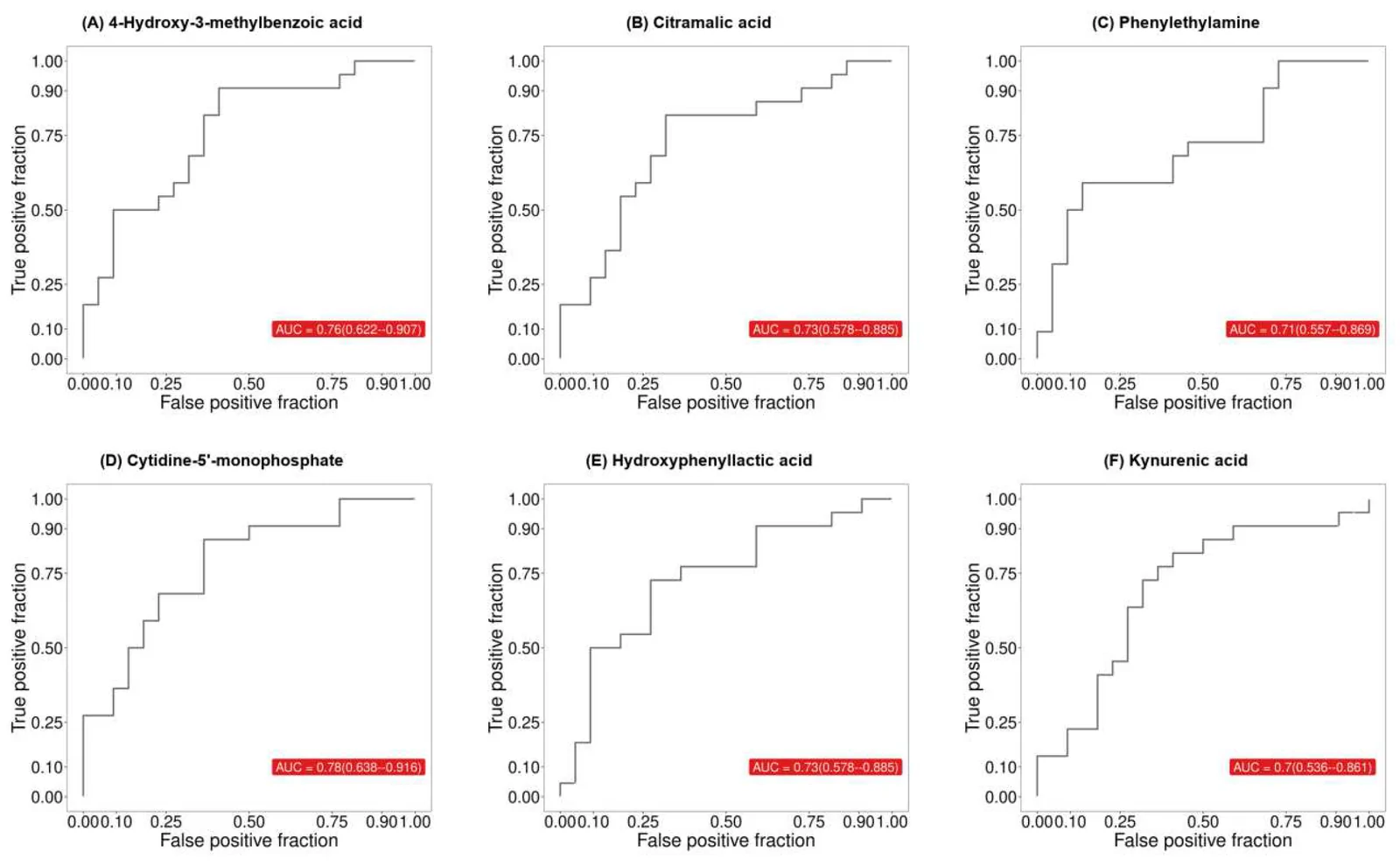
Descriptive ROC curves of representative exploratory candidate metabolites. Legend: (**A**) 4-hydroxy-3-methylbenzoic acid; (**B**) citramalic acid; (**C**) phenylethylamine; (**D**) cytidine-5′-monophosphate; (**E**) hydroxyphenyllactic acid; and (**F**) kynurenic acid. The AUC values summarize within-dataset separation and are presented descriptively because candidate identification and evaluation were performed in the same cohort.

Single-metabolite ROC curves were used to summarize within-dataset separation between emotional and reflex tears. Several candidates showed modest apparent separation, although no single metabolite provided strong discrimination. Salicylic acid also showed apparent separation but was excluded from the primary machine-learning analyses because the reflex-tear induction procedure may have contributed to this signal. The multivariable analyses therefore evaluated whether combinations of the remaining 22 candidates provided greater separation than individual metabolites (Figure 9). Because candidate identification and ROC evaluation were performed in the same cohort, the AUC values are presented as descriptive measures rather than estimates of clinical performance.

### 3.6. Exploratory Machine-Learning Discrimination Analysis

**Figure 10:**
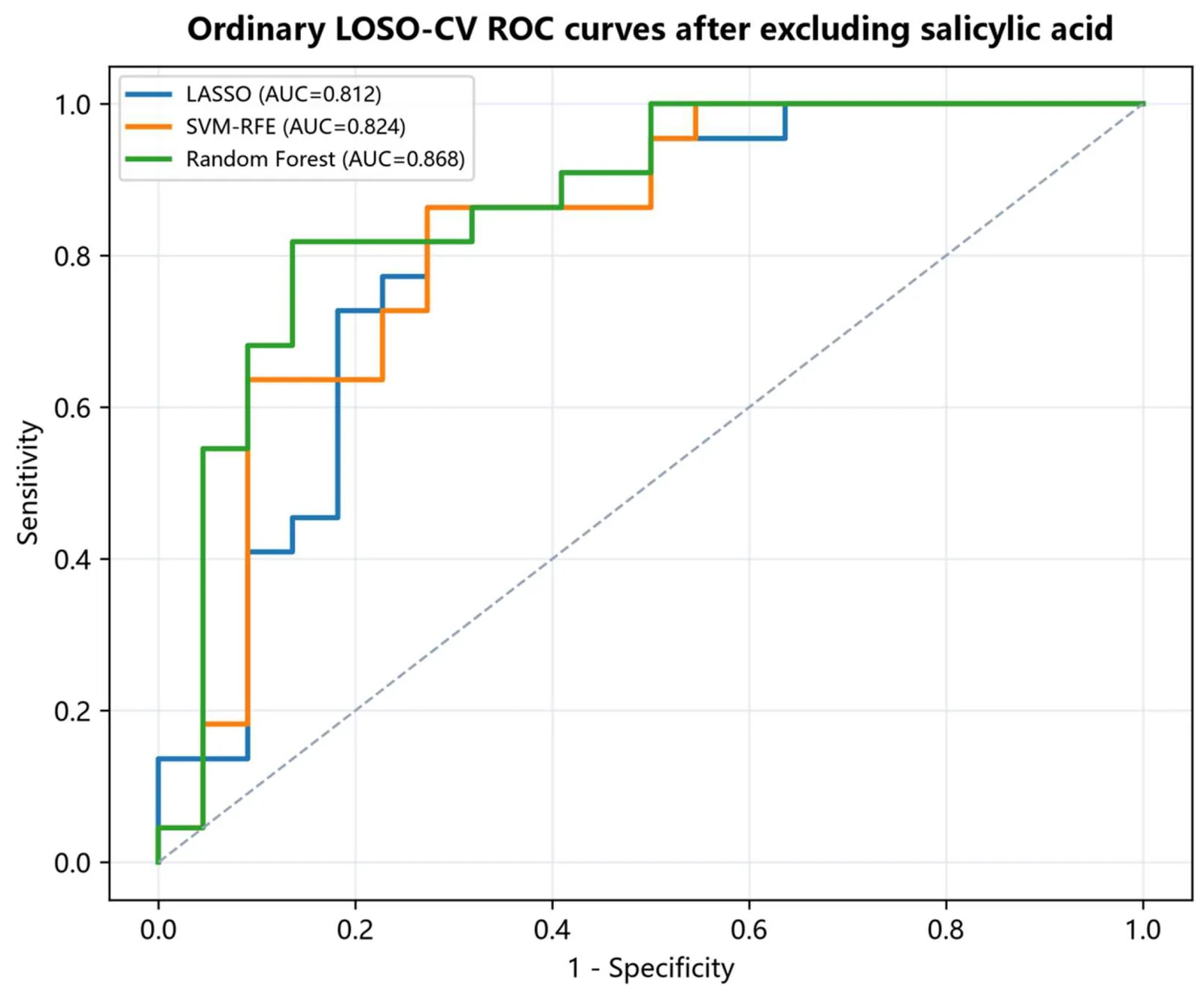
ROC curves of machine-learning models under ordinary LOSO-CV after exclusion of salicylic acid. Legend: The figure shows the ROC curves of LASSO, SVM-RFE, and random forest models evaluated under ordinary leave-one-subject-out cross-validation using the 22 candidate metabolites remaining after exclusion of salicylic acid. These results describe the discriminatory performance of the exploratory feature set defined using the full dataset, whereas the strict nested LOSO-CV results provide a more conservative estimate of model performance.

With salicylic acid excluded, 22 exploratory candidates entered the machine-learning analysis. In ordinary LOSO-CV, the AUC was 0.812 (95% CI, 0.707–0.913) for LASSO, 0.824 (95% CI, 0.696–0.934) for SVM-RFE, and 0.868 (95% CI, 0.769–0.957) for random forest. Random forest gave the highest AUC of the three methods (Figure 10).

**Figure 11:**
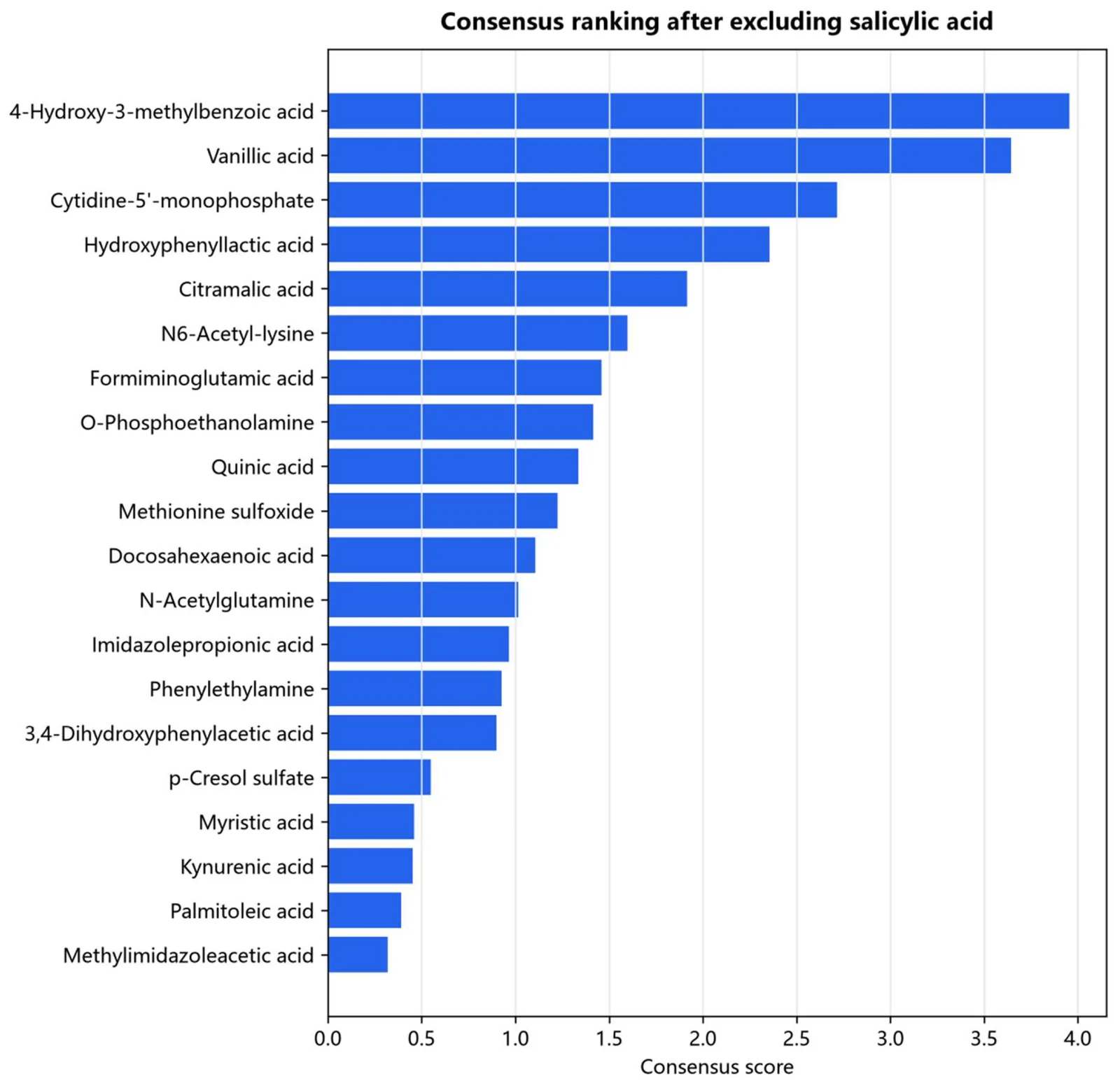
Consensus ranking of candidate metabolites by machine learning after exclusion of salicylic acid. Legend: The consensus ranking integrated LASSO selection frequency, frequency of inclusion among the top five SVM-RFE features, frequency of inclusion among the top five random forest features, and mean random forest importance. Salicylic acid was excluded from the primary ranking because of potential exogenous confounding. After its exclusion, 4-hydroxy-3-methylbenzoic acid, vanillic acid, cytidine-5′-monophosphate, and hydroxyphenyllactic acid ranked first through fourth, respectively, whereas docosahexaenoic acid and N-acetylglutamine ranked 11th and 12th, respectively.

Consensus ranking combined four pieces of information: LASSO selection frequency, frequency of appearing among the top five SVM-RFE features, frequency of appearing among the top five random-forest features, and mean random-forest importance. The first 10 metabolites were 4-hydroxy-3-methylbenzoic acid, vanillic acid, cytidine-5′-monophosphate, hydroxyphenyllactic acid, citramalic acid, N6-acetyl-lysine, formiminoglutamic acid, O-phosphoethanolamine, quinic acid, and methionine sulfoxide. Cytidine-5′-monophosphate ranked third; docosahexaenoic acid and N-acetylglutamine ranked 11th and 12th, respectively (Figure 11).

Fixed combinations were then built by adding metabolites in consensus-rank order. The ordinary LOSO-CV AUCs for Top 1 through Top 4 were 0.754, 0.762, 0.808, and 0.864. The Top 4 combination contained 4-hydroxy-3-methylbenzoic acid, vanillic acid, cytidine-5′-monophosphate, and hydroxyphenyllactic acid. Its AUC was 0.864 (95% CI, 0.756–0.957), with 100.0% sensitivity and 63.6% specificity.

We next evaluated the full feature-selection and modeling process with strict nested LOSO-CV. AUCs for the nested Top 1, Top 2, Top 3, and Top 4 pipelines were 0.632, 0.657, 0.725, and 0.698. The Top 3 pipeline gave the best balance of performance and parsimony, with an AUC of 0.725 (95% CI, 0.603–0.843), sensitivity of 90.9%, and specificity of 59.1% (Table 2).

These two analyses answer different questions. The fixed Top 4 result describes within-dataset discrimination for a fixed candidate combination selected using the full dataset, whereas nested LOSO-CV evaluates the complete feature-selection and model-building procedure. We therefore regarded the nested result as the more cautious estimate of out-of-sample performance.

## 4. Discussion

The paired design revealed a coherent set of within-subject metabolic differences between emotional and reflex tears. Twenty-three metabolites met the combined screening criteria, and the direction of change was preserved for all candidates in the Wilcoxon sensitivity analysis. After salicylic acid was excluded from modeling, 4-hydroxy-3-methylbenzoic acid, vanillic acid, cytidine-5′-monophosphate, and hydroxyphenyllactic acid were repeatedly prioritized across the machine-learning approaches. The contrast between the ordinary LOSO-CV result for the fixed Top 4 combination (AUC, 0.864) and the best strict nested LOSO-CV pipeline (AUC, 0.725) indicates that the metabolite pattern retained discriminatory information while also illustrating the optimism introduced when feature definition precedes cross-validation. No individual metabolite survived FDR correction; therefore, these results define a focused candidate set rather than a validated biomarker panel.

Once salicylic acid was removed from the primary modeling set, four metabolites repeatedly appeared near the top of the machine-learning rankings: 4-hydroxy-3-methylbenzoic acid, vanillic acid, cytidine-5′-monophosphate, and hydroxyphenyllactic acid. They point to several different areas of metabolism rather than one common pathway. Vanillic acid and 4-hydroxy-3-methylbenzoic acid are aromatic organic acids. Vanillic acid has been linked to aromatic compound metabolism and redox balance, whereas much less is known about the biological role of 4-hydroxy-3-methylbenzoic acid [9,10,11]. Its high ranking in this dataset therefore identifies it mainly as a candidate for follow-up rather than as a molecule with an established role in emotional tearing. Cytidine-5′-monophosphate belongs to pyrimidine nucleotide metabolism and participates in nucleic-acid turnover and related biosynthetic processes [12,13]. A change in its tear concentration could reflect altered nucleotide handling or cellular activity associated with secretion, although this remains speculative. Hydroxyphenyllactic acid is linked to aromatic amino acid metabolism and has also been discussed in relation to redox and microbial metabolism [14,15]. Taken together, these four leading candidates may reflect differences in several metabolic domains, including aromatic small-molecule metabolism, nucleotide-related processes, and redox-associated pathways. However, these interpretations remain preliminary and should not be considered evidence of a causal role in emotional tearing. Because none survived FDR correction, these interpretations are best treated as biological leads for future work.

Pathway mapping placed the 22 annotated candidates within histidine metabolism, fatty acid biosynthesis, tyrosine metabolism, ether lipid metabolism, and ubiquinone and other terpenoid-quinone biosynthesis. Histidine-related metabolism is linked to histamine-associated signaling processes [16,17], whereas tyrosine metabolism contributes to catecholamine biosynthesis [18,19]. Lipid-related and ubiquinone-associated pathways are relevant to membrane organization and redox regulation [20,21]. These connections provide biologically plausible context for the observed tear-type differences across amino-acid, lipid, and redox-related metabolism. Because the analysis was based on candidate metabolites rather than FDR-significant analytes, the mapped pathways represent priorities for targeted follow-up rather than direct evidence of pathway activation.

Interpretation of the machine-learning results depends strongly on how validation was performed. The ordinary LOSO-CV analysis started from candidates that had already been selected using the full dataset. Consequently, the participant left out for testing had indirectly influenced the earlier screening step, which can inflate apparent performance. Strict nested LOSO-CV was added to address this problem: within each outer fold, screening, ranking, feature selection, and model fitting were repeated using only the training participants. Under ordinary LOSO-CV, the fixed Top 4 combination reached an AUC of 0.864. In the nested analysis, the Top 3 and Top 4 pipelines reached AUCs of 0.725 and 0.698. These values are not two validations of the same fixed model. The first describes a fixed combination derived from the full dataset, whereas the second evaluates a complete selection-and-modeling workflow. For this reason, the nested result is the more conservative estimate and should carry greater weight when judging generalizability. Despite the use of participant-level cross-validation and strict nested LOSO-CV, overfitting remains an important concern because the number of evaluated metabolites exceeded the number of participants. The small sample size may affect the stability of feature selection and model estimates. Together, the ordinary and nested analyses provide complementary estimates of the discriminatory signal and define a compact candidate feature set for evaluation in independent cohorts.

A main advantage of the study is its within-subject design. Each volunteer provided both tear types, which reduced confounding from stable personal characteristics such as age, sex, diet, and baseline metabolism. The 600-MRM platform also allowed broad targeted measurement in a sample type that is available only in small quantities. Finally, using several machine-learning methods made it possible to look for recurring candidate patterns rather than relying on a single algorithm.

The study has several limitations. The cohort included only 22 healthy young volunteers, which limits statistical power and generalizability to older populations and patients with disease. No individual metabolite remained significant after FDR correction. Reflex tears were induced using an external stimulant whose exact composition could not be verified retrospectively; therefore, exogenous effects beyond the salicylic acid signal cannot be excluded. Nested LOSO-CV reduced the optimism associated with candidate definition using the full dataset, but neither the candidate metabolites nor the machine-learning models were externally validated. Circadian variation within the afternoon-to-evening sampling window and interindividual differences in emotional responses to film-induced crying may also have contributed to biological heterogeneity. Future studies should use larger independent cohorts and standardized, chemically defined or nonchemical tear-induction procedures.

In summary, this paired targeted-metabolomics study delineated within-subject metabolic differences between emotional and reflex tears and prioritized 23 candidate metabolites. Four metabolites were repeatedly ranked among the leading machine-learning features, while strict nested validation provided a more realistic estimate of their combined discriminatory signal. Although no individual metabolite survived FDR correction and no biomarker was validated in this cohort, the candidate set and associated pathway context provide a focused basis for independent validation in larger cohorts using standardized tear-induction procedures.

## Figures and Tables

**Table 1:** Baseline characteristics of the study participants (*n* = 22).

No.	Characteristic	Value
1	Age, years	24.6 ± 4.1 (range, 20–35)
2	Sex, n (%)	Male, 10 (45.5%); Female, 12 (54.5%)
3	Myopia, n (%)	14 (63.6%)
4	Spherical equivalent, D	−2.75 ± 1.68
5	Best-corrected visual acuity	Normal in both eyes (≥5.0)
6	Tear-film breakup time, s	9.8 ± 2.3
7	Schirmer I test, mm	14.7 ± 3.6
8	Contact lens use	None
9	Ocular disease history	None
10	Topical ophthalmic medication use	None

**Table 2:** Performance of machine-learning models under leave-one-subject-out cross-validation.

Model	Candidate Metabolites/ Feature-Selection Strategy	Validation Strategy	AUC	Sensitivity	Specificity
LASSO	22 candidate metabolites after exclusion of salicylic acid	Ordinary subject-level LOSO-CV	0.812	81.80%	72.70%
SVM-RFE	Top 5 features selected within each fold	Ordinary subject-level LOSO-CV	0.824	86.40%	72.70%
Random forest	22 candidate metabolites after exclusion of salicylic acid	Ordinary subject-level LOSO-CV	0.868	81.80%	86.40%
Fixed Top 3 combination	Top 3 metabolites in the consensus ranking	Ordinary LOSO-CV	0.808	59.10%	90.90%
Fixed Top 4 combination	Top 4 metabolites in the consensus ranking	Ordinary LOSO-CV	0.864	100.00%	63.60%
Nested Top 3 feature-selection pipeline	Top 3 features reselected using the training set only in each outer fold	Strict nested LOSO-CV	0.725	90.90%	59.10%
Nested Top 4 feature-selection pipeline	Top 4 features reselected using the training set only in each outer fold	Strict nested LOSO-CV	0.698	63.60%	72.70%

## Data Availability

The data supporting the findings of this study are available from the corresponding author, Jiahao Ye, upon reasonable request.

## Supplementary Materials

The following supporting information can be downloaded at: https://www.mdpi.com/article/10.3390/metabo16090607/s1, Figure S1. Principal component analysis (PCA) score plot of emotional and reflex tear samples. Figure S2. Orthogonal partial least-squares discriminant analysis (OPLS-DA) score plot of emotional and reflex tear samples. Supplementary Table S1. Relative standard deviations of internal-standard responses in quality-control samples. Supplementary Table S2. Detailed statistical results for the 23 exploratory candidate metabolites in emotional and reflex tears.

## Abbreviations

AUC, area under the receiver operating characteristic curve; CI, confidence interval; FDR, false discovery rate; HILIC, hydrophilic interaction liquid chromatography; KEGG, Kyoto Encyclopedia of Genes and Genomes; LASSO, least absolute shrinkage and selection operator; LC–MS/MS, liquid chromatography–tandem mass spectrometry; LOSO-CV, leave-one-subject-out cross-validation; MRM, multiple reaction monitoring; OPLS-DA, orthogonal partial least-squares discriminant analysis; PCA, principal component analysis; QC, quality control; ROC, receiver operating characteristic; RP, reversed-phase chromatography; SVM-RFE, support vector machine–recursive feature elimination; UHPLC, ultra-high-performance liquid chromatography.
