# Supplementary material for: Targeted Metabolomic Profiling of Emotional and Reflex Tears: A Paired Exploratory Study
Source: Metabolites. 2026 Aug 25;16(9):607. doi: 10.3390/metabo16090607 (PMC13608908; doi:10.3390/metabo16090607)
Supplement: Supplementary file 1 [file metabolites-16-00607-s001.zip › Supplementary Table S1.pdf]

Supplementary Table S1. Relative standard deviations of internal-standard responses in quality-control samples.

Platform	Internal standard	RSD (%)
RP Pos	pIS-01	3.88
RP Pos	pIS-02	6.02
RP Pos	pIS-03	1.6
RP Neg	nIS-01	2.13
RP Neg	nIS-02	0.86
RP Neg	nIS-03	0.83
HILIC Pos	pIS-01	3.02
HILIC Pos	pIS-02	8.66
HILIC Pos	pIS-03	1.89
HILIC Neg	nIS-01	16.65
HILIC Neg	nIS-02	6.34
HILIC Neg	nIS-03	12.12

Table note: RSD, relative standard deviation; RP, reversed-phase chromatography; HILIC, hydrophilic interaction liquid chromatography; Pos, positive-ion mode; Neg, negative-ion mode. RSD is reported as the coefficient of variation of internal-standard responses across the available quality-control measurements.
