# Supplementary material for: Targeted Metabolomic Profiling of Emotional and Reflex Tears: A Paired Exploratory Study
Source: Metabolites. 2026 Aug 25;16(9):607. doi: 10.3390/metabo16090607 (PMC13608908; doi:10.3390/metabo16090607)
Supplement: Supplementary file 1 [file metabolites-16-00607-s001.zip › Supplementary Table S2.pdf]

Supplementary Table S2. Detailed statistical results for the 23 exploratory candidate metabolites in emotional and reflex tears.

Metabolite	Fold change (Q/F)	Mean paired difference (Q−F)	95% CI lower	95% CI upper	Raw P value	BH-adjusted P value
Cytidine-5'-monophosphate	0.382857	-6.416203	-10.188585	-2.643821	0.001954	0.252361
4-Hydroxy-3-methylbenzoic acid	0.494459	-6520.715909	-11934.769538	-1106.662280	0.020568	0.283322
Glycerophosphocholine	0.572289	-19.320993	-35.602737	-3.039249	0.022271	0.283322
Salicylic acid	0.235097	-1698.464784	-3330.979416	-65.950153	0.042171	0.284448
O-Phosphoethanolamine	0.327602	-488.423875	-998.830835	21.983086	0.059763	0.302617
Vanillic acid	0.257766	-461.147391	-983.001441	60.706660	0.080299	0.332460
Docosahexaenoic acid	0.395949	-5905.001364	-12985.843550	1175.840823	0.097526	0.342337
Phenylethylamine	2.500757	14.360995	5.150628	23.571361	0.003898	0.252361
Formiminoglutamic acid	1.647981	72.161079	24.520168	119.801990	0.004832	0.252361
Kynurenic acid	1.881442	25.913673	8.751569	43.075776	0.004944	0.252361
Citramalic acid	2.278768	774.787167	235.502983	1314.071351	0.007015	0.252361
Myristic acid	2.242523	52866.004545	15593.928527	90138.080564	0.007651	0.252361
N6-Acetyl-lysine	2.457242	71.330465	21.026093	121.634837	0.007666	0.252361
Hydroxyphenyllactic acid	1.713365	155.612300	45.773168	265.451432	0.007711	0.252361
Palmitoleic acid	1.908169	129480.442727	37462.043536	221498.841919	0.008070	0.252361
Methionine sulfoxide	2.347382	1034.399333	277.306182	1791.492484	0.009778	0.258744
p-Cresol sulfate	2.812623	7679.445077	1798.453908	13560.436246	0.012954	0.280560
Quinic acid	2.128410	171.900883	27.076946	316.724819	0.022242	0.283322
3,4-Dihydroxyphenylacetic acid	1.767118	374.235468	37.307298	711.163639	0.031150	0.283322
Methylimidazoleacetic acid	2.226232	691.893850	49.720032	1334.067668	0.035990	0.283322
Imidazolepropionic acid	2.436856	35071.801818	2061.287614	68082.316023	0.038384	0.283322
Myristoleic acid	3.282589	169891.359045	-626.888284	340409.606375	0.050774	0.292267
N-Acetylglutamine	1.690601	92.711250	-7.457778	192.880278	0.067910	0.307384

Table note: Fold change was calculated as the ratio of the geometric mean concentration in emotional tears (Q) to that in reflex tears (F). The paired mean difference was calculated as Q−F. Raw P values were obtained using two-sided paired t tests. Benjamini–Hochberg-adjusted P values were calculated across all 344 metabolites retained for statistical analysis. CI, confidence interval; BH, Benjamini–Hochberg.
